# The growth of *Pediatric Rheumatology:* Pediatric Rheumatology European Society and the impact factor

**DOI:** 10.1186/1546-0096-10-19

**Published:** 2012-07-24

**Authors:** Charles H Spencer, Alberto Martini

**Affiliations:** 1 Nationwide Children's Hospital, Ohio State University, Columbus, OH, USA; 2 Department of Pediatrics, University of Genova, Genova, Italy

## 

We are pleased to announce that *Pediatric Rheumatology* (*PR*) has its first impact factor. The journal has just been given an impact factor of 1.44 this June after 5 years of association with the online publisher BioMed Central. This ranks our journal 59^th^ out of 113 pediatric journals and 22nd out of 29 rheumatology journals and we are just 5 years old (based on Impact Factor in the 2011 Journal Citation Reports) [1]. We have been listed on PubMed since 2007 and have applied for inclusion in MedLine. These are important steps in the growth and maturation of our journal. *Pediatric Rheumatology* is the only journal entirely devoted to our specialty with an impact factor and as an open access journal can therefore be reached by medical students, residents, and primary care physicians and specialists, as well as non-physicians and the general public all over the world.

We are delighted that the Pediatric Rheumatology European Society (PReS) has agreed to support this journal and align its prestigious name and reputation with us as of January 1, 2013. For the next 5 years the Society will provide financial support to *PR* to maintain a competitive price for authors to publish who need assistance-many authors do not.

Open access journals represent the future of scientific publications and many University and Research Centers in the Western world already support their researchers by covering the costs of publication in these type of journals. The American National Institutes of Health and the British Wellcome Trust require their grantees to publish open access within 12 and 6 months respectively, and as the open access movement gathers momentum [2,3], funding bodies are faced with the issue of effectively ensuring compliance to these policies [4]. Many Universities support publication in our Publisher’s journals, with BioMed Central Membership accounts providing either a full or partial discount for their researchers [5]. Moreover, authors from many low-income countries [6] can publish without charge. For those authors who do not have the above mentioned discounts and waivers, publication costs in open access journals are at present higher with respect to the other journals because of the lack of subscription fees. The financial support of PReS will indeed allow competitive publication costs for authors that do not have access to the above mentioned facilities. As the open access model is gradually more supported by governments and granting organizations [7], as appears likely over the next decade, the financial support of PReS may be needed less.

Meanwhile we hope that our *PR* journal will become worthy of many important pediatric rheumatology publications as the journal grows, and our impact factor increases. The link between *PR* and PReS does fit with one important goal of PReS and all pediatric rheumatology organizations. We wish to provide a useful, common forum for pediatric rheumatologists all over the world and stimulate the growth of pediatric rheumatology, especially in growth areas such as Africa and Asia, where our subspecialty is just beginning to mature. Our open access model should help interested physicians and other professionals learn more about our field and the diseases we care for. Ultimately, we want to do our utmost to do what we can to improve medical care for children with rheumatic and musculoskeletal diseases everywhere and help each child. Many of us in the Western World come from relatively privileged circumstances. We at *PR* believe it is our duty and responsibility to do what we can for every child with JIA, lupus, dermatomyositis, and other rheumatic diseases, no matter what their status and situation. We hope this effort to support *PR* will in turn help these children we care about. Recent educational efforts of PReS, such as our Mumbai, India symposium in November 2012, also address our need to spread the gospel of pediatric rheumatology.
